# Clinical Ethics and Law Teaching: An Important Challenge for International Medical Education Partnerships

**DOI:** 10.1007/s40670-025-02372-1

**Published:** 2025-04-12

**Authors:** Anjali Rajendra Gondhalekar, Mohammed Ahmed Rashid

**Affiliations:** 1 Institute of Health Sciences Education, Barts and the London School of Faculty of Medicine and Dentistry, Queen Mary University of London, The Garrod Building, Turner Street, London, E1 2 AD UK; 2 London School of Hygiene and Tropical Medicine, 15 - 17 Tavistock Pl, London, WC1H 9SH UK

**Keywords:** Collaborative/peer-to-peer, International medical education, Ethics/attitudes

## Abstract

There is an increasing commitment to building educational partnerships with institutions delivering healthcare professionals education globally. Projects with higher educational institutions in the Global South have become well established and whilst much of the educational content developed is easily translatable to partners across continents, one challenge is navigating contrasts that exist in ethics and law within international medical education. In this commentary, we reflect on the challenges faced when developing such medical educational resources and how we must avoid a ‘one size fits all’ approach through our collaborative efforts.

The increasing frequency and importance of international partnerships in medical education reflects a growing recognition of the value of global collaboration in training future healthcare professionals. These partnerships offer students access to diverse learning environments, helping them develop a broader understanding of global health issues. As healthcare becomes more interconnected, institutions are seeking international collaborations to share resources, research, and clinical training, benefiting both students and faculty. These partnerships often involve the sharing of curricular and assessment materials across partner institutions in different countries and contexts. Many areas within medical education transfer and fit well when applied to international institutions and these include the introduction of material on basic sciences, clinical theory, and clinical skills teaching, which are more easily transferable to our curricula abroad. Whilst there are some contrasts in epidemiology and local health priorities, we feel that the most interesting differences exist within the fields of clinical professionalism, ethics, and law. Due to the contrasting ethical, cultural, and religious perspectives in international institutions, it is essential that we avoid adopting a ‘copy and paste’ approach [1].

UK clinical ethics and law teaching forms a well-established part of the undergraduate curriculum for many allied healthcare professional courses including medicine, nursing, pharmacy, and dentistry. In the UK, professional bodies such as the General Medical Council (GMC) have set out key guidelines including ‘Outcomes for Graduates’ which outline the road map for the coverage of ethics and law within the undergraduate medical curriculum nationally. In many Western nations, ethics and law is such a well-established specialist area of study that many institutions provide specific MSc programmes in this field, which are designed for allied healthcare professionals. This further highlights the vast level of understanding and confidence in healthcare ethics and law education within the UK. Globally, ethics and law is recognized as an important curriculum area in health professions’ education and is even outlined in the World Federation for Medical Education (WFME) standards. Nevertheless, it could be argued that there is some disparity in the implementation of ethics and law in global medical curriculums when compared to more Western healthcare profession’s education systems [2].

When working on international projects, it is easy to assume a neo-imperial stance and assume that UK practice supersedes international practices. This can in part be due to the well-developed codes of practice that organisations such as the Nursing and Midwifery Council (NMC), the General Dental Council (GDC), and GMC provide us, which the Global South may lack the infrastructure to develop. At an institutional level, it is essential that we consciously acknowledge the important cultural nuances across different nations, as well as the significant contrasts in religious practises and variations in legal systems [3, 4], which inform the delivery of this content. This inevitably means that when collaborating with international teams, we must avoid blindly utilising the frameworks that we lean on within the UK system, but instead work in collaboration with our institutional partners in order to adapt healthcare education material, which is in keeping with local ethics, law, and religious codes of practice. One of many areas of the medical educational curriculum where such flexibility is essential includes the delivery of women’s health teaching, especially within topics such as sexual health counselling and contraception as well as in antenatal and postpartum care. In order to bridge this gap in our understanding of these differences, we must ensure that we take the opportunity to learn in greater depth about our partners’ local practices and clinical approaches. In this way, we can work to identify the best method of supporting the development of ethics teaching as part of health professions’ education in terms of not only curriculum content but also within assessment material produced, thereby achieving constructive alignment across these core concepts. Through this combined approach with our partners, we hope to contribute to the preparation of these upcoming allied healthcare professionals’ educational development so that they feel well equipped to approach these challenges of ethics in their day-to-day practice both in their own countries as well as globally.

International health professions education partnerships are a fascinating intellectual challenge. As we continue to work with international institutions globally, we continue to harness the learning opportunities afforded to better understand global clinical practices and viewpoints within health professions education [5]. Whilst we feel that in many areas, drawing on practices from developed countries can be valuable, we must also acknowledge that it is important to appreciate the cultural identities of partnering institutions, whose local knowledge and practice must be prioritized when developing syllabus content to cover.
